# Nitrate Respiration in *Thermus thermophilus* NAR1: from Horizontal Gene Transfer to Internal Evolution

**DOI:** 10.3390/genes11111308

**Published:** 2020-11-04

**Authors:** Mercedes Sánchez-Costa, Alba Blesa, José Berenguer

**Affiliations:** 1 Center for Molecular Biology Severo Ochoa (CBMSO), Autonomous University of Madrid-Spanish National Research Council (UAM-CSIC), 28049 Madrid, Spain; mercedes.sanchez@cbm.csic.es; 2 Department of Biotechnology, Faculty of Experimental Sciences, Francisco de Vitoria University, 28223 Madrid, Spain; alba.blesa@ufv.es

**Keywords:** denitrification, evolution, thermophile, horizontal gene transfer, nitrate respiration, PacBio sequencing

## Abstract

Genes coding for enzymes of the denitrification pathway appear randomly distributed among isolates of the ancestral genus *Thermus*, but only in few strains of the species *Thermus thermophilus* has the pathway been studied to a certain detail. Here, we review the enzymes involved in this pathway present in *T. thermophilus* NAR1, a strain extensively employed as a model for nitrate respiration, in the light of its full sequence recently assembled through a combination of PacBio and Illumina technologies in order to counteract the systematic errors introduced by the former technique. The genome of this strain is divided in four replicons, a chromosome of 2,021,843 bp, two megaplasmids of 370,865 and 77,135 bp and a small plasmid of 9799 pb. Nitrate respiration is encoded in the largest megaplasmid, pTTHNP4, within a region that includes operons for O_2_ and nitrate sensory systems, a nitrate reductase, nitrate and nitrite transporters and a nitrate specific NADH dehydrogenase, in addition to multiple insertion sequences (IS), suggesting its mobility-prone nature. Despite nitrite is the final product of nitrate respiration in this strain, the megaplasmid encodes two putative nitrite reductases of the *cd1* and Cu-containing types, apparently inactivated by IS. No nitric oxide reductase genes have been found within this region, although the NorR sensory gene, needed for its expression, is found near the inactive nitrite respiration system. These data clearly support that partial denitrification in this strain is the consequence of recent deletions and IS insertions in genes involved in nitrite respiration. Based on these data, the capability of this strain to transfer or acquire denitrification clusters by horizontal gene transfer is discussed.

## 1. Introduction

Many prokaryotes use denitrification as their main energy generation respiratory mechanism in the absence of oxygen or when this preferential electron acceptor is scarce in the environment [1]. The canonic process has been well described in excellent reviews [2,3,4,5] and involves four steps of successive reductions (nitrate > nitrite > nitric oxide > nitrous oxide > dinitrogen), encoded by the corresponding membrane or periplasmic reductases (Nar/Nas, Nir, Nor, Nos) expressed either by a single organism or by different ones sharing a common environment, allowing, as a whole, the displacement to the atmosphere of soluble nitrogen species [2,6,7]. However, in many cases, the last step of the pathway is less efficient and nitrous oxide, a powerful greenhouse gas, is the final product of the process, being accumulated in the atmosphere [8,9,10,11].

Indeed, the denitrification process has been studied in great detail for many years but using essentially model proteobacteria [3,12,13,14]. Nonetheless, there is also available relevant information about this process in Gram-positives (Firmicutes) [15], and in few extremophiles, both archaea [16] and bacteria [17]. Among these last ones, denitrification genes and actual functionality have been described for phylogenetically diverse species, many of which are thermophiles [18]. The genus *Thermus* is one of the few ancestral phylogenetic groups for which the denitrification process has been studied to some extent (reviewed in [17]).

This genus belongs to the ancient clade *Deinococcus–Thermus* and includes hundreds of moderate to extreme thermophile strains, isolated from both natural and man-made environments, currently grouped into 27 species (https://lpsn.dsmz.de/genus/thermus), among which *Thermus aquaticus* and *Thermus thermophilus* are the most studied. The genus itself was originally defined as aerobic, and, actually, many strains are unable to grow in the absence of oxygen. However, anaerobic growth with nitrogen oxides has been described for few strains of the genus, ones growing only with nitrate and others able to grow too with nitrite as electron acceptors, leading to the final production of nitrous oxide in some of these strains [17,19,20].

In addition to being used as frequent sources of enzymes of biotechnological interest, few isolates of the genus *Thermus* have been employed as study model according to their easy adaptation to fast growth under laboratory conditions, in both complex and mineral media, and their highly efficient natural competence system [21], which has allowed the development of quite a complete set of genetic tools [20,22,23,24,25].

Among these laboratory-adapted models, the *T. thermophilus* HB8, HB27 and NAR1 strains have been extensively used. The first two were the first *Thermus* strains for which complete genome sequences were available [26]. Both are non-fermentative, obligate aerobes that harbor a highly syntenic 1.8 Mbp chromosome, with less than 6% of differential genes [27]. They also share a megaplasmid named pTT27 of 232,605 and 256,992 bp for HB27 and HB8 strains, respectively, allocating the highest gene diversity, with variable regions containing differential strain-specific genes. The HB8 strain contains another 81,151 bp megaplasmid (pVV8) that has been lost in many laboratory-adapted derivatives [28], and a smaller (9322 bp) plasmid named pTT8, of higher copy number, both absent in the HB27 strain. While the HB8 strain has been used mainly for structural genomic analysis (http://www.thermus.org/e_index.htm), the employment of HB27 strain has been focused on cell-physiology analysis and as a host for biotechnological applications due to its higher transformability compared to HB8, reaching constitutive DNA uptake rates up to 40 kbp per cell and second [29]. In contrast to the HB8 and HB27 strains, *T. thermophilus* NAR1 strain is able to grow anaerobically with nitrate, with final production of nitrite, which is accumulated in the medium. The genes encoding this property have been cloned and sequenced following classic methods, allowing the identification of the main enzymes implicated in this process (reviewed in [17]). Further partial sequencing of this strain based on Illumina technology allowed the confirmation of these data, although the structure of the coding region within the genome or the presence of genes putatively involved in further steps of the denitrification process could not verified due to the apparent massive presence of insertion sequences, which generated a quite fragmented genome. The recent application of new single-molecule sequencing methods based on the PacBio technology [30], in combination with previous Illumina data, has allowed the generation of a full assembled genome for this strain. The analysis of this consensus sequence, described for the first time in this work, reveals a quite complex evolutionary history of the denitrification pathway in *T. thermophilus* NAR1.

## 2. Materials and Methods

### 2.1. DNA Preparation and Sequencing

*T. thermophilus* NAR1 (TthNAR1) is a strain which was originally mistaken with the strain HB8. It was renamed as NAR1 once the sequence of HB8 was available and the presence of specific genes, including the ability to grow on nitrate, was revealed [17]. TthNAR1 was grown overnight at 65 °C under aerobic conditions in 20 mL of Thermus broth [31]. Genomic DNA isolation was carried out following the standard protocol of phenol-chloroform, including a RNAse A treatment. After quality control assessment on agarose gel and quantification by ND1000 Nanodrop spectrophotometer, sample was submitted to whole genome sequencing, which was performed following 2 approaches: PacBio RS II SMRT cells (PacBio) and mate-pair Illumina (Solexa). PacBio sequencing was done for single reads (1X 16,500) at NSC Centre (Norway), and Illumina sequencing was carried out following the mate-pair system (Miseq, read type 2 × 36 − 251) at Secugen (Scientific Park, Campus UAM, Madrid, Spain).

### 2.2. Genome Sequence Analysis and Assembly

Illumina reads were trimmed using Trimmomatic [32], and quality sequence analysis were performed over reads using FastQC software [33]. Sequence alignment and preliminary assembly was performed using Newbler Assembler and CLC Genomics Workbench 4.0.3 (CLCBio). Contigs were aligned and sorted against reference genome using an in-house script of BLAST. Scaffold sequences were obtained using Scaffold builder (https://github.com/metageni/Scaffold_builder) which sorted the 307 contigs generated by draft sequencing, filled gaps and small overlaps following alignment with Needleman–Wunsch algorithm. This was also done using LAST aligner (http://last.cbrc.jp/).

PacBio sequencing results encompassed 60,929 reads with a medium size of 11,306 bp which were de novo aligned using HGAP3 (Pacific Biosciences, SMRT Analysis Software v2.3.0) and compared to the one provided by the sequencing center using QUAST (http://bioinf.spbau.ru/quast). Reads were aligned with pbalign following BLASR method [34] and coverage of each alignment was performed with GenomeCoverageBed (http://bedtools.readthedocs.io/en/latest/content/tools/genomecov.html) and visually represented using GNUplot (http://gnuplot.info/).

As reported elsewhere [35,36,37], after the assembly and annotation of the PacBio sequences, several indels errors were spotted by manual inspection and comparison with Illumina-based sequences. Therefore, PacBio assembly had to be corrected using the Illumina aforementioned reads. Briefly, Illumina reads were aligned to the PacBio assembly with Bowtie2 aligner (http://bowtie-bio.sourceforge.net/bowtie2/index.shtml) and detection of indels was performed using Pilon [38] and PacBio utilities. A polished consensus genome assembly of the PacBio information supplemented with the Illumina sequences led to the selection of 4 contigs corresponding to the 4 replicons found in TthNAR1 strain. Coverage of each alignment was performed with GenomeCoverageBed and visually represented using DNAplotter (https://www.sanger.ac.uk/tool/dnaplotter/).

### 2.3. Annotation of TthNAR1 Replicons

The scaffold genome obtained from Illumina reads was annotated using RAST [39]. PacBio genome assembly annotation was carried out using 3 software tools, following different automated approaches in order to ensure consonant functional annotation. PROKKA [40], RAST [39] and AAMG [41] were executed and the results obtained were compared using BEACON [42] and then filtered to avoid duplications. Finally, functional annotation of the final consensus genome assembly integrating the PacBIO genome assembly supplemented with short-accurate Illumina reads was executed using PROKKA. Genomic maps of the detected replicons were visually represented with DNAplotter (http://www.sanger.ac.uk/science/tools/dnaplotter).

The sequences employed in this study were extracted from the whole-genome project 506,402 which have been deposited in EMBL/GenBank under the accession number PRJEB29203 (https://www.ncbi.nlm.nih.gov/bioproject/506402). Data from this PacBio *de novo* genome assembly described herein (GCA_900604845.1) were verified using Illumina sequences from a prior genome sequencing project of this strain (unpublished). Sequences used can be checked here (https://www.ncbi.nlm.nih.gov/bioproject/PRJEB29203/).

### 2.4. Phylogenomic Assessments

*T. thermophilus* NAR1 consensus genome assembly was compared to fully assembled genomes from other available genomes of *Thermus* spp. at the NCBI database using an *in-house* script for BLAST, and genes’ synteny was checked by filtering for coverage higher than 80%. A multiple alignment using Mauve [43] for chromosome replicon and Gepard (http://cube.univie.ac.at/gepard) and MumMmer (http://mummer.sourceforge.net/) for the plasmids was executed, the latter visually represented by dotplots. Presence of denitrification genes was verified using the raw Illumina reads and homologs were searched using BLAST (NCBI).

## 3. Results and Discussion

### 3.1. Sequence of the TthNAR1 Strain

**Figure 1:**
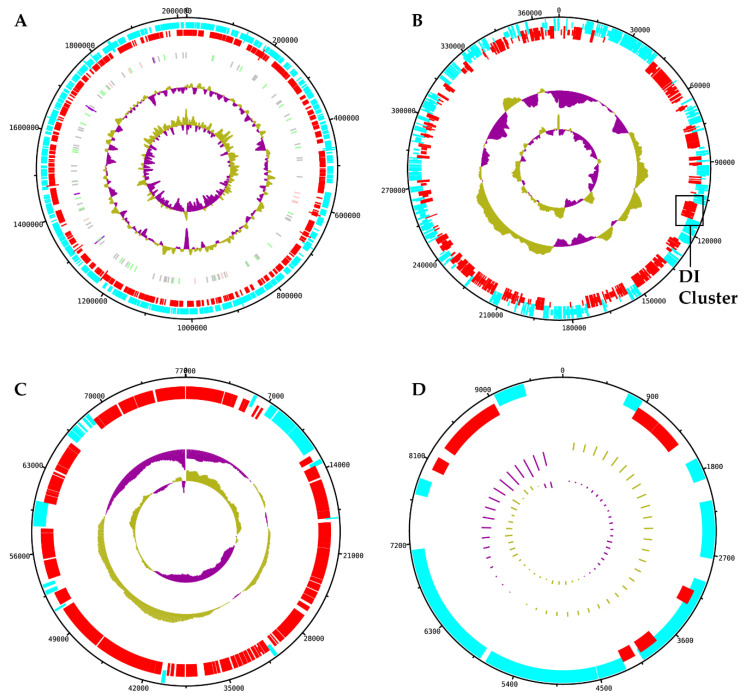
Genome of TthNAR1. Circular maps of the four replicons detected in TthNAR1 strain, elaborated using DNAPlotter using the consensus genome assembly achieved from supplementing PacBIO sequences with Illumina data. This strain harbors a highly conserved 2 Mbp chromosome (**A**) and plasmids of different sizes: a pTT27-like megaplasmid of 370 kbp (**B**) and two smaller plasmids of 77 kbp (**C**) and 9.8 kbp (**D**). These last two plasmids have little relation to other plasmids in the gene bank, as described in Figure S1. Localization of the nitrate and nitrite respiration cluster is indicated as DI cluster in the pTT27-like megaplasmid (**B**).

The PacBio-generated sequences of TthNAR1 strain shows a GC content similar to other *Thermus* strains (68.79%) and higher similarity to TthJL18 genome (84.5% homology) followed by that of TthHB8 strain (78.7%). The genome was divided into four contigs that could be circularized in the corresponding replicons (Figure 1).

**Figure 2:**
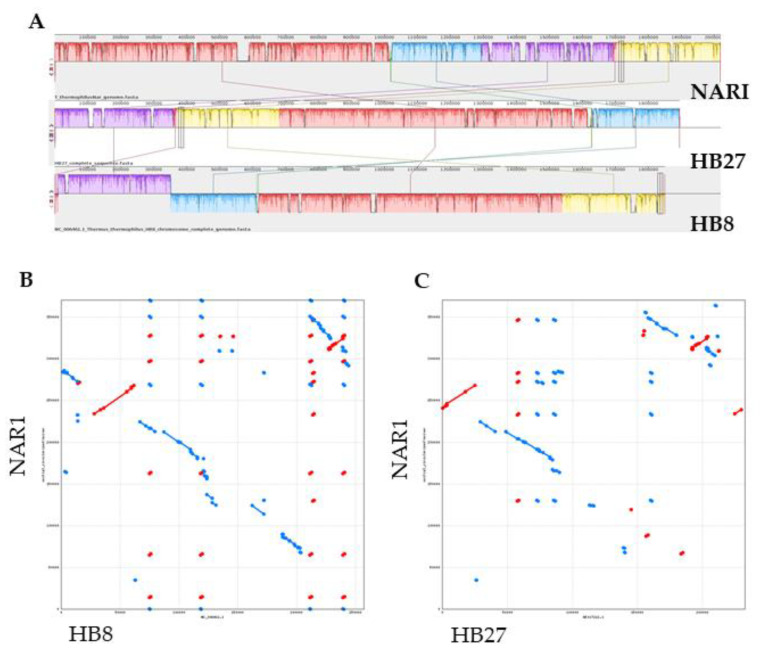
Synteny of TthNAR1 replicons with other Tth strains. (**A**) Alignment of (top to bottom) Tth strains NAR1, HB27 and HB8 chromosomes with Mauve (no connection lines). The sequence has been divided in four section colors corresponding to syntenic regions to facilitate the visualization. Dotplots of TthNAR1 370 kbp megaplasmid against pTT27 megaplasmids found in HB8 (**B**) and HB27 (**C**).

The larger contig, of 2,019,399 bp, corresponds to the chromosome which shares most of its genes with the chromosome of the HB8 and HB27 strains. Moreover, the order of chromosomal genes of TthNAR1 is similar to that of TthHB27, whereas an inversion is detected in the chromosome of HB8 (Figure 2A). The second replicon in size corresponds to a megaplasmid of 370,488 bp which shares many common genes with pTT27, despite the presence of strain-specific genes and two large inversions (Figure 2B,C). Based on the coverage, this plasmid has a similar copy number to that of the chromosome (a ratio of 1.3:1). A smaller plasmid of 77,040 bp was also detected in TthNAR1, which showed no similarities with pTT27 but actually shared few genes that are present in plasmid pVV8, the third plasmid of TthHB8, present in old stocks of the strain, but not in laboratory-adapted derivatives [28]. However, several regions of this plasmid were shared with plasmids pTTJL1801 and pTHTHE1601 from the strains TthJL18 and TthSG0.5, respectively (Figure S1D,E). Interestingly, the sequence coverage reveals a lesser copy number for this plasmid than that of the chromosome (0.38:1), suggesting that it could be lost in part of the population. Finally, a smaller plasmid with a copy number apparently similar to that of the chromosome (1:1.3) is also present in TthNAR1 (Table 1). This plasmid shows scarce similarity to the small plasmid pTT8 described in TthHB8 (Figure S1F).

A further manual analysis of the PacBio genome revealed the presence of mutations that truncated genes encoding for essential proteins. As an example, the DNA gyrase genes *gyrA* and *gyrB* were truncated at three positions, leading to two and three ORFs in what should correspond to the subunits A and B of the DNA gyrase, respectively. The comparison of these sequences with that of Illumina reads did not show any of these apparent mutations, supporting the existence of errors in the PacBio assembly. Interestingly, these apparent mutations were not randomly distributed along the genome, but concentrated and reiterative at specific points, where more than 90% of the PacBio reads (coverage above X300 except for pTTHNP3) contained the same error (a G deletion) and, thus, were incorporated by the software to the corresponding consensus PacBio sequence. By contrast, 90% of Illumina reads at the same point (coverage X50) showed no interruptions in the coding frame and encoded the corresponding potentially functional wild type proteins.

Due to the identification of these systematic errors, a thorough genome-wide search for differences between PacBio-based and Illumina-based reads in TthNAR1 was carried out. This analysis revealed the existence of 2927 deletions along the PacBio genome and a single insertion in PacBio sequences respect to the Illumina counterpart that essentially affected homopolymers of 5–6 units of G or C. This number of errors corresponds to around one deletion every 1000 bases, thus suggesting the presence of a great number of artifactual apparent mutants (1297 ORFs) in the initial PacBio sequence annotation of the strain. Interestingly, these mutations were not distributed along all the G/C homopolymers types but affected only 1.64% of those present in the genome, suggesting the existence of additional sequence features that could be affecting the activity at these points of the PacBio single molecule reading system. Nonetheless, a search for putative sequence pattern using as template a 20 bp sequence upstream and downstream from the systematic reading errors did not reveal any significant sequence pattern consensus that could justify it.

It is also relevant to note that a similar effect of systematic errors in PacBio-based sequences compared to Illumina reads was detected for *Mycolicibacteriun hassiacum* [44], another genome harboring high G + C content, although the number of systematic errors found in this genome was one tenth of that observed for TthNAR1 (481 in a 5 Mbp genome, roughly one in 10,000 bases). In addition, this case, no apparent sequence pattern around the points where the reads systematically failed could be found. In any case, these results clearly support that a combination of technologies is needed to obtain an accurate genome sequence, at least for organisms showing high G + C content, and the users of the databases should be aware of the methodologies followed to obtain the published sequences.

In the TthNAR1 case, the errors detected in the PacBio sequences were corrected with the Illumina reads, and the final genome was assembled and annotated, showing no present indels in any essential gene. In the corrected sequence, the analysis of the genome (Materials and Methods) showed the presence of six copies of the ribosomal RNAs, 51 tRNA genes and 2767 CDS coding for putative proteins (Table 1). In addition, using the IS finder program with default search values, many ISs, some of them putatively active and others truncated, were found along the four replicons. In addition, four CRISPR arrays were found, allocated in the megaplasmid pTTHNP4, and two putative ones were also detected in pTTHNP3.

### 3.2. The Nitrate Reductase Gene Cluster

**Figure 3:**
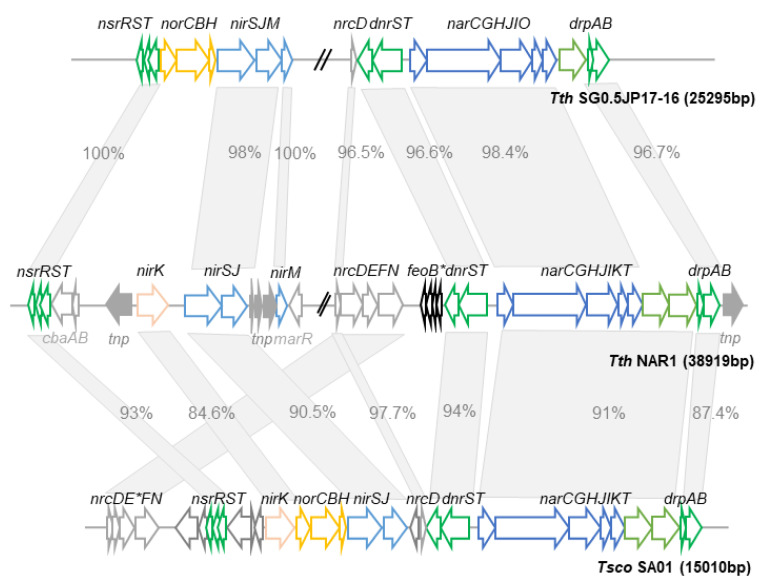
Comparison of the denitrification clusters of *TthNAR1* and other *Thermus* spp. Genetic context of nitrate and nitrite respiration genes found in *TthNAR1*, showing homolog genes and its order in the closely related strains *TthSG05 JP17-16* and *T. scotoductus* SA-01 (*Tsco* SA01). Genetic maps were performed according to fully assembled genomes available at NCBI database. Grey empty arrows represent genes not related with nitrate/nitrite respiration. Filled grey arrows represent transposases or fragments of transposases (indicated as *tnp*). Genes anchored with an asterisk indicate truncated sequences (pseudogenes). Percentages of identities are indicated within the grey boxes and length of the arrows is proportional to the size of the CDS.

The gene cluster coding for nitrate respiration (*nar*) is localized between positions 140,059 and 158,930 of the pTTHNP4 megaplasmid. This cluster includes 15 ORFs encoding functional proteins; for most of them, their participation has been experimentally verified at different steps of nitrate respiration (Figure 3). A pseudogene harboring three frameshifts that originally encoded a putative iron transporter is also found within the cluster, suggesting its lost in a host context where this element is efficiently transported.

Proteins required for nitrate respiration are expressed in four transcriptional units (Figure 3). The largest one is the *narCGHJIKT* operon which codes for the conserved components of the nitrate reductase (NarGHI), a chaperone (NarJ) needed for the insertion of the diMGD cofactor in NarG, a cytochrome C (NarC) absent in Nar operon in mesophiles, and two nitrate/nitrite transporters (NarK and NarT). A four-gene operon, *nrcDEFN*, encodes a NADH dehydrogenase that shows structural similarities to succinate dehydrogenase and is transcribed only under anoxia in the presence of nitrate. Finally, two bigenic operons, *dnrST* and *drpAB*, encoding regulatory proteins that respond to oxygen (DnrS and DnrT) or to nitrate (DrpA and DrpB), are located upstream and downstream, respectively, of the *narCGHJIKT* operon.

When the sequence of the *nar* cluster from *TthNAR1* was compared to that found in other nitrate respiring strains of *Thermus* spp., such as *T. thermophilus* SG0.5JP17-16 (*TthSG*) or *T. scotoductus* SA01 (*Tsco*), two main differences became clear. On the one hand, the dispensability of the NADH dehydrogenase system (Nrc): in *TthSG* it is absent except for the *nrcD* ferredoxin and a pseudogene deletion form of *nrcE*; and in *Tsco* it is separated into two group of genes, *nrcD* on one side and *nrcDF* in the other, also lacking *nrcE* (Figure 3). On the other hand, the nitrate/nitrite transporters, where the tandem NarK–NarT is found in *TthNAR1* and *Tsco*, are replaced by a single transporter of a different MFS subfamily (NarO) in *TthSG*. Interestingly, NarO can surrogate the pair NarKT when expressed in *trans* in denitrifying derivatives of the strain *T. thermophilus* HB27 [45].

By contrast, the sequence and synteny of the genes encoding the nitrate reductase (*narCGHJI*) and that of the regulators (*dnrST* and *drpAB*) is highly conserved, supporting that they constitute the core enzyme and the regulatory control systems for nitrate respiration. Interestingly, this region can be horizontally transferred to aerobic strains of *Thermus* spp. such as *T. thermophilus* HB27 by conjugation or by natural competence, allowing the new host to grow anaerobically with nitrate [17].

### 3.3. The Respiratory Nitrate Reductase (NR) of TthNAR1 and Other Thermus spp.

As commented above, the core of the nitrate respiration cluster includes five genes conserved in all the nitrate respiring strains of *Thermus* spp. so far described, instead of the four genes typically described for the NR of mesophilic model organisms [46]. The additional gene is expressed at the 5′ extreme of the mRNA and encodes a membrane-bound periplasmic cytochrome with two heme C groups [47]. Mutants lacking this protein are unable to grow anaerobically under nitrate conditions and further biochemical analysis revealed that they produced a soluble and immature form of NarG [48], the protein subunit where nitrate is reduced, suggesting the requirement of NarC for the maturation and membrane attachment of the enzyme. A similar behavior was found for mutants lacking NarI, the bi-heme B membrane cytochrome, conserved in all the respiratory nitrate reductases characterized so far. Furthermore, bacterial two-hybrid assays supported the existence of strong interactions between the two cytochromes NarC and NarI, as well as among NarI, NarH and NarG, whereas the NarJ chaperone was only shown to interact with NarG [48]. All these data clearly support the existence of a heterotetrameric nitrate reductase that contains a periplasmic cytochrome C as an additional subunit, absent in the NR reductases described in other model bacteria.

**Figure 4:**
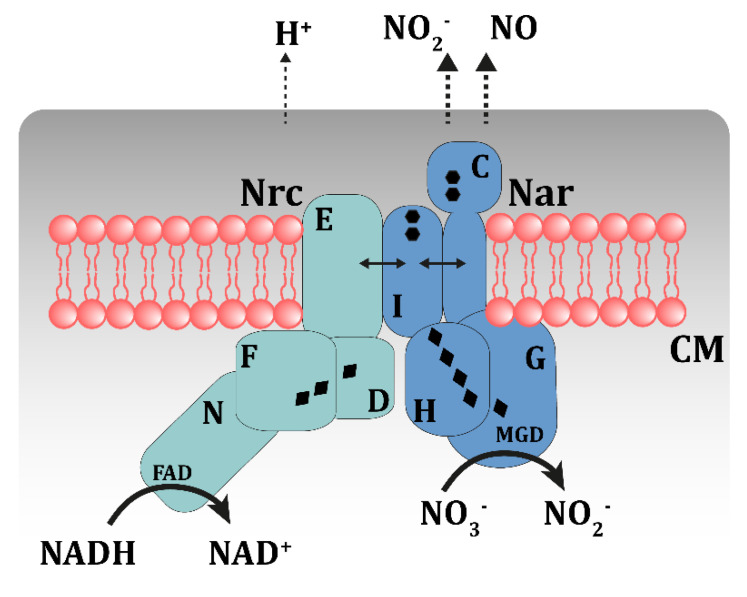
A putative nitrate respirasome in *TthNAR1*. This scheme shows the likely structure of the nitrate respirasome, including the Nrc NADH dehydrogenase complex and the nitrate reductase. Activity of the complex will separate protons. Interactions detected by two-hybrids assays are indicated as double arrowheads lines. Heme groups are indicated as black hexagons and iron sulfur clusters as diamonds. The role of the NarC component of the NR as electron transporter towards NOR and NOS enzymes is indicated as discontinuous arrows.

In addition to being required for the synthesis and maturation of an active NR, the putative participation of NarC cytochrome in the electron transport within nitrate respiration is not well defined yet. However, it could probably be working as an electron-transport branching system, deriving electrons to other acceptors in case of nitrate scarcity. In this sense, it is noteworthy to mention the presence of a very similar gene cluster (to the *Thermus* one) in nitrifying bacteria such as *Nitrobacter hamburguensis*, where the encoded enzyme function as nitrite oxidoreductase, involved in the oxidation of nitrite to nitrate by the NarG-homolog, and the electrons generated going through the cytochrome B (NarI homologue) to the periplasmic NarC homolog, with final destination in complex III or complex IV (the cytochrome oxidase) [1]. Interestingly, in denitrifying strains of *T. thermophilus* such as TthPRQ16, the lack of NarC makes the cell unable to grow with nitrite or NO as electron acceptors [49] in a context where the expression of the complex III is inhibited, suggesting the involvement of NarC as an electron bridge towards the periplasmic nitrite reductase or the membrane nitric oxide reductase (Figure 4). In this context, complementation experiments showed the greater relevance of the distal heme C group in the process [49]. If this were the actual role of the fourth component of the *Thermus* NR, it should be deduced that the ability to respire nitrite and NO has to be the rule and not the exception in nitrate respiring strains. In other words, nitrate respiration has to be biochemically linked to nitrite respiration in odds to an efficient denitrification performance.

### 3.4. A Putative Nitrate Respirasome in the NAR1 Strain

The presence of a NADH dehydrogenase bound to the membrane through a protein complex in the *nar* cluster of TthNAR1 suggests the putative existence of a “nitrate respirasome” in this strain (Figure 4). The *nrcDEFN* operon encodes a ferredoxin (NrcD) that in bacterial two-hybrid assays strongly interacts with the polytopic membrane protein NrcE [50]. The cytoplasmic C terminus of NrcE concomitantly interacts with an iron-sulfur containing protein (NrcF) similar to that found in succinate:quinone oxidoreductases, and with NrcN, a flavoenzyme that shows type-II like NADH dehydrogenase activity when overexpressed and purified from *E. coli* [50,51]. In addition, in *TthNAR1*, bacterial two-hybrid assays revealed strong interactions between NrcF and NrcD. At physiological levels, mutants in *nrcE, nrcF* or *nrcN* (*nrcD* was too small so as to get insertion mutants) showed slower growth rates than the wild type strain under nitrate respiration conditions, and the membrane fractions from mutants lacking any of these proteins showed a significantly decreased NADH oxidation capacity in the presence of nitrate compared to the wild type performance. All these data, together with those figures regarding the parallel transcriptional induction of the *nrcDEFN* and *narGDHJIKT* operons under nitrate respiration, pointed to the existence of a highly efficient transfer of electrons between both complexes, likely through the formation of a nitrate respiratory supercomplex (Figure 4). It is important to note that the existence of such a complex has not been biochemically proven yet, but bacterial two-hybrid assays strongly support the existence of interactions between both protein complexes through their membrane units NrcE and NarI, which could ease the exchange of electrons between them, either directly or through the menaquinones.

### 3.5. Regulation of Nitrate Respiration in TthNAR1

As in other model bacteria, the synthesis of the enzymes for nitrate respiration depends on two concomitant environmental signals: the decrease in oxygen below a threshold level and the presence of nitrate in the growth medium [3]. In most of these model systems, oxygen is sensed through a transcriptional regulator of the cAMP family, named FNR, that contains a [4S-4Fe] iron–sulfur cluster as the oxygen sensor [52]. Upon oxidation of the cluster, FNR behaves as a monomer and lacks affinity for its binding sites. In the genome of *TthNAR1*, there are no FNR homologs that could play such a role, so oxygen sensing has to be displayed through a different mechanism. On the other hand, due to its membrane impermeability, in most denitrifying Proteobacteria, nitrate sensing is carried out by two-component systems of the NarX/NarL family, where the nitrate sensory component, NarX, dimerizes upon detection of nitrate at the periplasmic side of the membrane, and a phosphorylation system leads to the phosphorylation of the response regulator of the NarL family, which subsequently binds to a heptameric sequence, found in diverse repeats upstream the regulated genes, which also usually contain binding sites for the FNR dimers [3,53]. As with the oxygen sensing, *TthNAR1* does not encode homologs to NarX/NarL system despite its responsiveness to nitrate, supporting the existence of a different class of nitrate sensors in this ancient bacterial group.

The identification of the sensory systems for oxygen and nitrate within the *nar* cluster was the consequence of horizontal gene transfer experiments towards the aerobic *TthHB27* strain [31,54]. In these experiments, the oxygen and nitrate sensors were transferred along the nitrate reductase operon within an approximately 30 kbp DNA fragment. Subsequently, it was shown that mutants lacking any of the Dnr proteins (DnrS and DnrT, formerly RegA and RegB in [55]), encoded immediately upstream of the *nar* operon (Figure 3), were defective in anaerobic growth with nitrate due to their requirement for the transcription of the *narCGHJIKT* operon. However, only DnrT (and not DnrS) was required for the transcription of the *nrcCEFN* operon, supporting the existence of functional differences among the role of these proteins. Further, these assays demonstrated that DnrT (a transcriptional factor of the CRP family) was insensitive to oxygen—actually it has no iron–sulfur cluster—or nitrate signals and was acting in a concentration-dependent manner through the binding to sites located approximately at −43 position from the starting site of the regulated promoters (i.e., CCTTCACCTTACTCCTTGACCCCGGTCAT in Pnrc), where it was used to recruit the RNA polymerase [55]. In contrast, the conformation of DnrS was sensitive to the presence of oxygen, as revealed by its increased sensitivity to proteases under aerobic conditions. Despite these data showing DnrS oxygen sensitivity, the actual oxygen sensor in DnrS is not known, but it is likely dependent on the GAF domain found at its N-terminal region. Besides, its putative DNA binding capability could not be assayed, likely due to the mentioned sensitivity to oxygen. Despite these uncertainties yet to be solved, it seems clear that the *dnrST* operon, conserved in all the nitrate reductase clusters described for *Thermus* spp., is involved in oxygen detection. In this context, it is also worth mentioning the role of DnrT, not only as a transcription activator factor for all the promoters involved in the expression of genes required for nitrate respiration, but also as repressor of the pbc1 promoter, responsible for the expression of the complex III in *T. thermophilus*, suggesting that another electron transporter replaces this complex during anaerobic growth.

**Figure 5:**
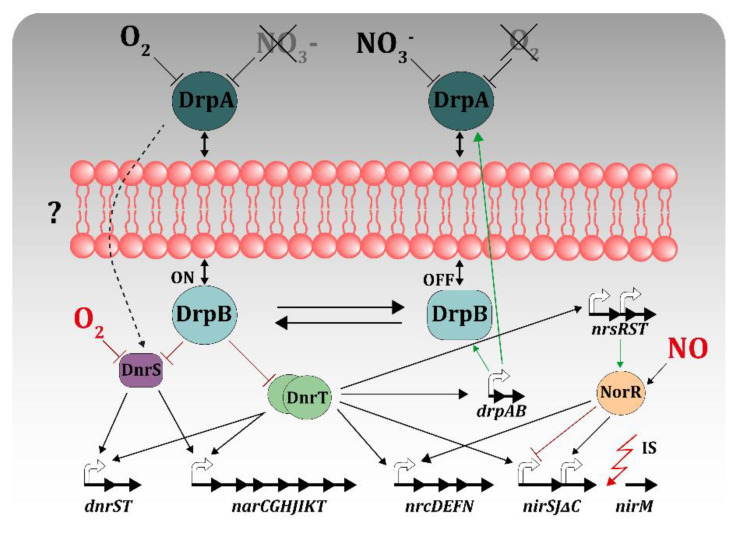
Regulatory circuitry controlling nitrate respiration. DnrS is the oxygen sensor of the system, being active in its absence and allowing its own expression and that of cotranscribed gene *dnrT*, as well as acting in the transcription of the nar operon. DnrT is a general activator needed for transcription of all the operons for nitrate respiration as well as to transcribe *nir*, *nor* and *nrs* operons needed for nitrite respiration in denitrifying strains of Tth, a process in which DnrS is also needed. NorR is a transcription factor that stimulates transcription of *nrc, nirJ* and its own operon, but represses nirS promotion in the presence of NO. DrpB activity on transcription is likely to occur through interaction with the master regulator proteins in the absence of nitrate, and releases this activity by an unknown mechanism (?) when DrpA detects nitrate in the periplasm (see the text for details).

The nitrate detection system was also difficult to assess because of the high sensitivity of the TthNAR1 strain to this compound, which required the use of rich media prepared with the same yeast extract and bactopeptone stocks, as some of these commercial products had enough nitrate amounts in their composition to activate the system in the absence of additional nitrate. In any case, the nitrate sensory system was also genetically linked to the nitrate respiration cluster, as observed in the horizontal gene transfer experiments, and the focus was set on the *drpAB* operon located immediately downstream of the *narCGHJIKT* operon in all the *Thermus* spp. so far sequenced [17]. By employing specific antisera in Western blot assays, it was found that DrpA was periplasmic, whereas DprB was cytoplasmic, although attached to the membrane [56]. Despite the lack of knowledge of the actual mechanism of nitrate sensing by these proteins, it is clear that the transcription of the *nar, nrc* and *dnrST* operons solely depends on the absence of oxygen in mutants lacking both DrpA and DrpB proteins, and is completely signal independent—i.e., constitutive—in the absence of DrpB, suggesting a repressor role for DrpB protein, in contrast to the transcription activator role played by phosphorylated NarL [56]. However, as the sequence of DrpB does not show any putative DNA binding motif, its action could be likely dependent on an interaction with the master regulators DnrS or/and DnrT (Figure 5), in a way similar to what was proposed for the nitrate/oxygen co-sensing protein NreA of *Staphylococcus aureus* [57]. Concomitantly, the expression of DrpA and DrpB was dependent on the concentration of DnrT, but not on DnrS, supporting the existence of an induction/repression circuit that keeps the concentration of the four regulators under control, allowing the re-setting of the system [56]. A speculative model for the regulatory circuit that controls nitrate respiration in TthNAR1 is shown in Figure 5, in which the presence of oxygen and absence of nitrate keeps DrpB active to inhibit DnrS and/or DnrT, whereas presence of nitrate along absence of oxygen inactivates it, allowing DnrS and DnrT to activate the nitrate respiration clusters.

### 3.6. Genes for Nitrite Respiration in TthNAR1

Nitrite respiration in denitrifying strains such as *TthPRQ25* and *TthPRQ16* depends on the expression of the *nirSJM* operon, which codes for the periplasmic cd1 type nitrite reductase (NirS), a protein involved in the synthesis of the *heme d* cofactor (NirJ), and a periplasmic cytochrome C_550_ (NirM) proposed to act as electron transporter towards NirS [58,59].

On the other hand, the presence of a periplasmic cytochrome C (NarC), as a fourth subunit of the nitrate reductase of *Thermus* spp. discussed above, suggests that the capability of this enzyme to replace the complex III during further steps of the denitrification pathway [49] is a general property that allows the transfer of electrons to compatible external acceptors in case of nitrate scarcity. Actually, in complete denitrifying strains such as *TthPRQ25*, nitrite accumulates in the medium until nitrate becomes scarce, and then the former is used as electron acceptor, likely employing the NarC component of the NR as surrogate to the complex III, repressed under these conditions. This suggests that nitrate and nitrite respiration are not simultaneous but are functionally linked. The genomic proximity of the genes encoding both processes in those denitrifying strains has already been sequenced, and the requirement of the master regulators DnrS and DnrT for nitrite respiration [59] clearly support the existence of a complete denitrification island. This denitrification island can be one-step transferred to the aerobic *TthHB27* strain, leading to the selection of a denitrifying derivative (*TthHB27d*) [17]. Interestingly, the nitrate respiration cluster of the nitrate respiring *TthNAR1* strain can control the nitrite respiration cluster from other strains [17], supporting that inability of *TthNAR1* to use nitrite happens to be an anomaly.

Indeed, the availability of the complete genome sequence of *TthNAR1* allowed us to confirm this unicity. Executing BLASTP searches with the *TthSG* NirS, NirJ and NirM protein sequences, we found homologs (98–100% of identity) for the three proteins in *TthNAR1* pTTHNP4 megaplasmid (positions 170,194 to 171,804; 168,964 to170,112 and 167,260 to 167,694, respectively), allocated near the nitrate respiration cluster, as if they were part of an old denitrification island. In addition, in that same region, *TthNAR1* encodes a putative nitrite reductase of the Cu-Nir type (NirK), similar (84.6% of identity) to that of *T. scotoductus* [60]. The presence of two types of nitrite reductase (*cd1* and Cu-types) in a single organism is quite unusual, but it strongly suggests that a recent ancestor of *TthNAR1* strain displayed nitrite respiration capability.

The reasons by which *TthNAR1* cannot use nitrite despite the presence of genes coding for two types of nitrite reductases seemed clear when a detailed analysis of the sequences was carried out. On the one hand, a transposase pseudogene of the IS256 family, fragmented into three ORFs (TTHNP4_186/187/188), is found inserted between the *nirJ* and the *nirM* genes, which could limit or abolish the expression of the latter (Table 2, Figure S2). On the other hand, the putative Cu-Nir (NirK, TTHNP4_0191) lacks 49 N-terminal amino acids respect of that of *Tsco* due to the insertion of another IS (IS982-like, TTHNP4_192). The lost region of NirK includes the signal peptide needed for periplasmic secretion of this protein and also the putative promoter signals required for its expression, supporting that the protein is not synthesized at all.

In conclusion, the expression of Cu-Nir (NirK) has been lost by the insertion of an IS, and the *nirJSM* cluster has been divided into two fragments, with high chances of no expression of the cytochrome *nirM*. Further analysis suggests that these insertion events would have likely occurred a long time ago, as the corresponding transposases are pseudogenes due to the presence of several mutations within them.

### 3.7. Genes for NO Respiration

Nitrite respiration generates NO and leads to the concomitant requirement for an active NO reducing system, either to use it as electron acceptor for respiration, or at least to remove this toxic compound in an effective manner. To this purpose, close to the nitrite reductases’ sequences, all the denitrifying *Thermus* strains so far studied show an heterotrimeric nitric oxide reductase [54,61] encoded by the *norCBH* operon and a NO-sensory system encoded by the *nrsRST* operon [62].

The genome of TthNAR1 does not encode the *norCBH* operon, neither in the region close to the nitrite reductase genes nor in other regions of the genome. Actually, no residual coding regions corresponding to these genes were located at the expected positions, suggesting that these genes were lost a long time ago, or that another unrelated enzyme develops this function. However, homologs to the NO-responsive regulator NsrR and its associated proteins NsrS and NsrT form a cluster almost identical in sequence to that of *TthPRQ25* or *TthSG*, supporting that the system is functional for NO detection despite the absence of the *norCBH* genes. In this context, it is interesting to note that, immediately upstream of the *nsrRST* operon, and encoded in the same transcriptional sense, two genes are found that code for proteins annotated as cytochrome-oxidase subunits CbcB (TTHNP4_195) and CbcA (TTHNP4_196). Keeping in mind the similarities and common phylogenetic origins between NOR and cytochrome oxidases, and the presence of these two genes in a denitrification genomic context, it is tempting to speculate that these enzymes actually use NO as substrate. It is also worth noting the presence of a similar *cbcBA* cluster immediately upstream the sequence of NirK in *T. scotoductus*, although in *TthNAR1,* an IS, is integrated in between them, suggesting a putative inactivation of the corresponding promoter.

## 4. Conclusions

The current availability of whole genome sequences from many *Thermus* spp. has allowed the identification of genes involved in different metabolic processes. Nonetheless, in the case of denitrification, the existence of complete and partial denitrifying strains as well as aerobic ones has been puzzling. The employment for many years of the *TthNAR1* strain as a model to study nitrate respiration demanded a complete and verified genome in order to achieve a better understanding, and re-interpretation when needed, of those experimental results.

Upon sequencing of the *TthNAR1* strain with the PacBio technology, we identified systematic errors in the reads that affected many genes, actually absent when the Illumina sequences were overlayed, so a significant conclusion from these bioinformatic analysis arises: double method sequencing should be mandatory for whole genome sequencing of high G + C content organisms. Once the sequences of *TthNAR1* were corrected, we were able to assemble four circular contigs, one of which, a megaplasmid with a scaffold shared with the pTT27 megaplasmids of the aerobic strains HB27 and HB8, encoded the enzymes involved in nitrate respiration, a metabolic trait extensively studied in this *TthNAR1* strain. Moreover, thanks to the availability of the whole consensus sequence, we found a group of genes encoding enzymes for nitrite respiration upstream the nitrate respiration cluster in what constitutes a whole denitrification island. This element includes genes for nitrite respiration and a regulator for NO sensing, but no genes encoding a NOR enzyme were found, despite their presence in other denitrifying strains of the *Thermus* genus. In addition, we found a panoply of IS elements, apparently inserted in a random manner, that either separated genes within a cluster (*nirM* from *nirSJ*) or inactivated them (*nirK*) (Table 2), suggesting that the ability to respire nitrite was lost in an ancestor of the *TthNAR1* strain and discarding the hypothesis that suggested the acquisition of the nitrate reductase cluster by an aerobic ancestor to render the nitrate respiring strain.

## Figures and Tables

**Table 1:** Summary of features of the consensus *TthNAR1* replicons. CDS, RNA and tRNA were identified with PROKKA, and IS using the ISFinder database.

Contig	Bases	Coverage	CDS	Rep. Region (CRISPR)	rRNA	tRNA	IS
Chromosome	2,021,843	339.8	2220	0	6	51	70
pTTHNP2	9799	448.2	12	0	0	0	19
pTTHNP3	77,135	130.5	106	2 (questionable)	0	0	102
pTTHNP4	370,865	464.9	429	4	0	0	100

**Table 2:** Putative transposases located within the denitrification cluster of TthNAR1 strain. Typology of the transposases have been deduced according to the ISfinder database (https://isfinder.biotoul.fr/).

Position	ORF	IS-Type
163912–162710	TthNP4_176 + TthNP4_177	ISTth4
167718–168215	TthNP4_186 *	ISTth4
168356–168670	TthNP4_187 *	ISTth4
168631–168915	TthNP4_188 *	ISTth4
173562–174395	TthNP4_192	IS982-like

* Transposases that can belong to a unique ORF.

## Supplementary Materials

The following data are available online at https://www.mdpi.com/2073-4425/11/11/1308/s1, Figure S1: Plasmids synteny. Figure S2: Presence of transposases within the denitrification cluster.
