# Supplementary material for: Nitrate Respiration in Thermus thermophilus NAR1: from Horizontal Gene Transfer to Internal Evolution
Source: Genes (Basel). 2020 Nov 4;11(11):1308. doi: 10.3390/genes11111308 (PMC7694296; doi:10.3390/genes11111308)
Supplement: Supplementary file 1 [file genes-11-01308-s001.zip › Supplementary Files.docx]

Supplementary Files to

Nitrate respiration in *Thermus thermophilus* NAR1: from horizontal gene transfer to internal evolution

Mercedes Sánchez-Costa ^1^, Alba Blesa ^2^ and José Berenguer ^1,^*

^1^ Centro de Biología Molecular Severo Ochoa (CBMSO), Universidad Autónoma de Madrid-Consejo Superior de Investigaciones Científicas, Madrid 28049, Spain; mercedes.sanchez@cbm.csic.es; jberenguer@cbm.csic.es;

^2^ Department of Biotechnology, Faculty of Experimental Sciences, Universidad Francisco de Vitoria, Madrid, 28223, Spain; alba.blesa@ufv.es

***** Correspondence: jberenguer@cbm.csic.es; Tel.: +34-911964498


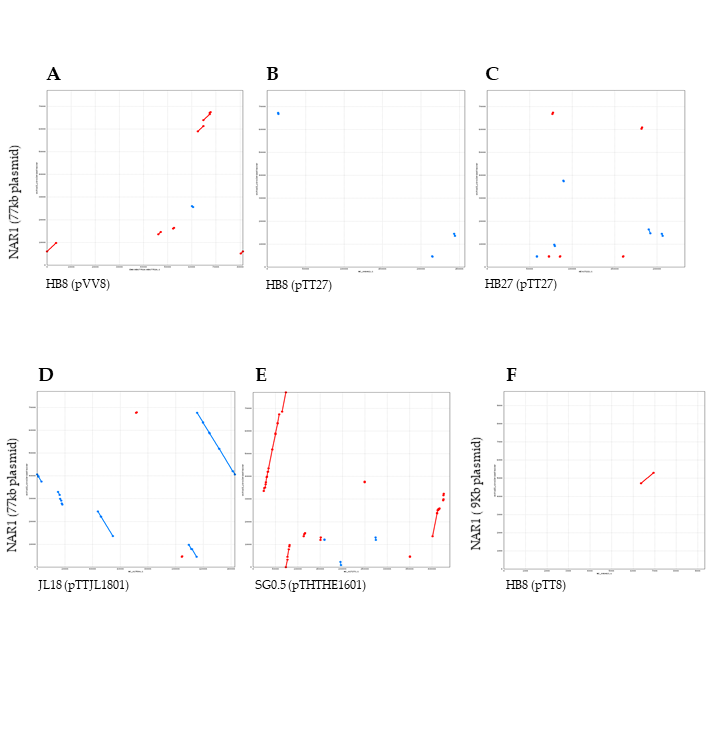


**Supplemental 1. Synteny analysis of small plasmids from TthNAR1** Dotplots of NAR1 77 kbp megaplasmid against TthHB8 plasmids, pVV8 (A) and pTT27 (B), TthHB27 pTT27 megaplasmid (C), TthJL18 pTTJL1801 megaplasmid (D) and TthSG0.5 pTHTHE1601 megaplasmid (E). An additional dotplot of the 9 kbp plasmid from TthNAR1 genome aligned to HB8 cryptic plasmid pTT8 is included (F).

**
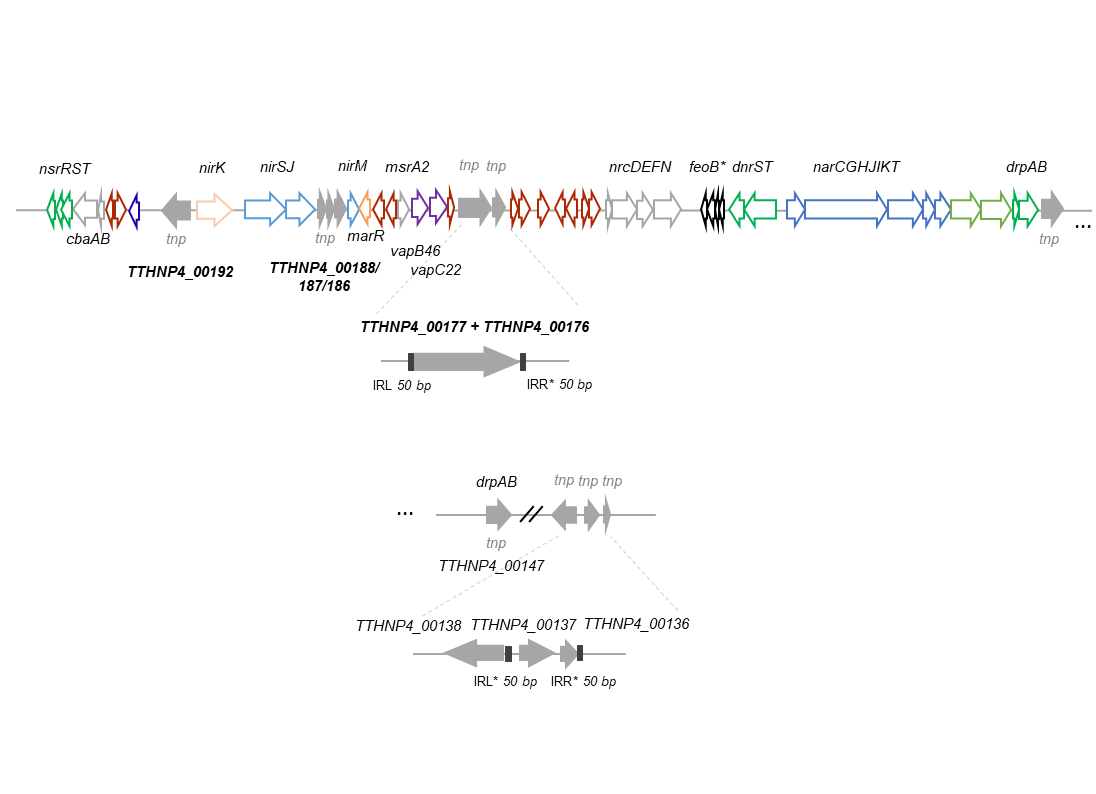
**

**Supplemental 2. Presence of transposases and transposition events in *Thermus thermophilus* NAR1 denitrification cluster.** Genetic context of the denitrification cluster found in TthNAR1, showing the localization of transposases (arrows filled in grey), their loci and the identification and length of the inverted repeats (IRs) of the complete most relevant ones.
