# Supplementary figures and images for: Nitrate Respiration in Thermus thermophilus NAR1: from Horizontal Gene Transfer to Internal Evolution
Source: Genes (Basel). 2020 Nov 4;11(11):1308. doi: 10.3390/genes11111308 (PMC7694296; doi:10.3390/genes11111308)

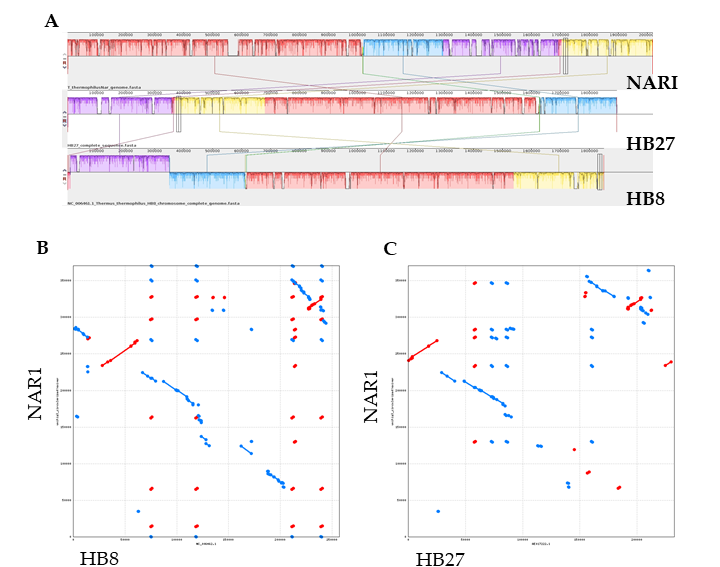

Supplement: Supplementary file 1 [file genes-11-01308-s001.zip › Figure2.tif]

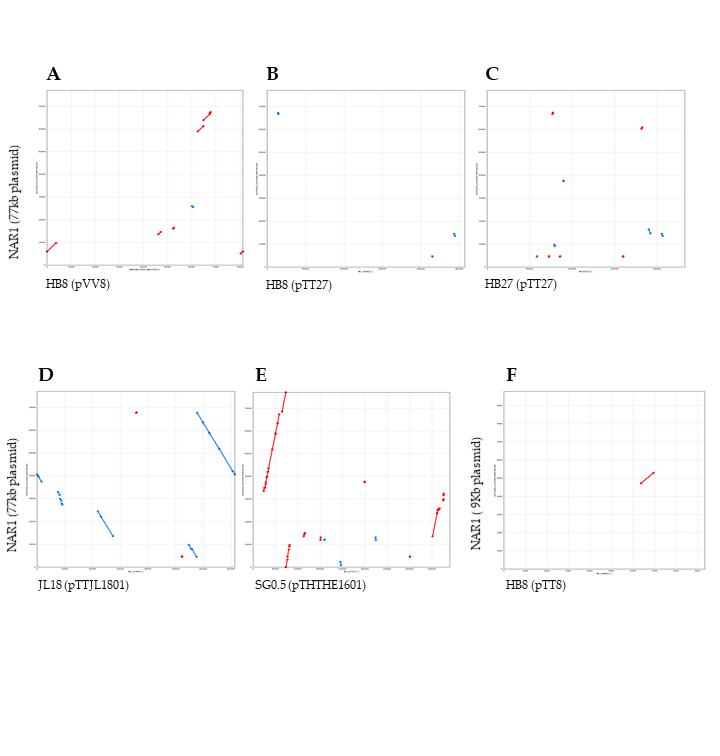

Supplement: Supplementary file 1 [file genes-11-01308-s001.zip › FigureS1.tif]
